# Lactose Hydrolysis in Milk and Dairy Whey Using Microbial *β*-Galactosidases

**DOI:** 10.1155/2015/806240

**Published:** 2015-10-26

**Authors:** Michele Dutra Rosolen, Adriano Gennari, Giandra Volpato, Claucia Fernanda Volken de Souza

**Affiliations:** ^1^Laboratório de Biotecnologia de Alimentos, Programa de Pós-Graduação em Biotecnologia, Centro Universitário UNIVATES, Avenue Avelino Tallini 171, P.O. Box 155, 95900-000 Lajeado, RS, Brazil; ^2^Curso de Biotecnologia, Instituto Federal de Educação, Ciência e Tecnologia do Rio Grande do Sul (IFRS), Câmpus Porto Alegre, Porto Alegre, RS, Brazil

## Abstract

This work aimed at evaluating the influence of enzyme concentration, temperature, and reaction time in the lactose hydrolysis process in milk, cheese whey, and whey permeate, using two commercial *β*-galactosidases of microbial origins. We used *Aspergillus oryzae* (at temperatures of 10 and 55°C) and *Kluyveromyces lactis* (at temperatures of 10 and 37°C) *β*-galactosidases, both in 3, 6, and 9 U/mL concentrations. In the temperature of 10°C, the *K. lactis β*-galactosidase enzyme is more efficient in the milk, cheese whey, and whey permeate lactose hydrolysis when compared to *A. oryzae*. However, in the enzyme reaction time and concentration conditions evaluated, 100% lactose hydrolysis was not reached using the *K. lactis β*-galactosidase. The total lactose hydrolysis in whey and permeate was obtained with the *A. oryzae* enzyme, when using its optimum temperature (55°C), at the end of a 12 h reaction, regardless of the enzyme concentration used. For the lactose present in milk, this result occurred in the concentrations of 6 and 9 U/mL, with the same time and temperature conditions. The studied parameters in the lactose enzymatic hydrolysis are critical for enabling the application of *β*-galactosidases in the food industry.

## 1. Introduction

Milk is recognized by its nutritional value as it presents in its composition an elevated concentration of calcium and proteins of biological value. It also has bioactive peptides with a protective function in human health, such as antibacterial, antiviral, antifungal, antioxidant, antihypertensive, and antithrombotic action [1, 2].

Dairy whey, such as cheese whey and whey permeate, is widely used in the elaboration of new products due to its lactose, proteins, vitamins, and dietary minerals compositions [3–5]. In addition to their nutritional properties, cheese whey proteins present the capacities of solubility, formation of foam, water absorption, gelatinization, and emulsification. These technological properties of the cheese whey proteins confer beneficial characteristics to foods [6].

However, the lactose present in milk, in dairy products and in whey from dairy industries, limits the consumption of these products by individuals who are lactose intolerant. Intolerance to this sugar is a result of the reduction in the *β*-galactosidase enzyme levels in intestinal walls. This enzyme is present in mammals during the breast-feeding period; however, for most of them, the *β*-galactosidase activity decreases after the said period, which characterizes primary hypolactasia and brings about symptoms of lactose intolerance. This disorder affects about 70% of the world adult population [7–10]. According to Mattar and de Campos Mazo [7], in Brazil, the disease occurs in 57% of the white population and 80% of black individuals, reaching 100% of the population of Japanese descent.

The food industry has been seeking to develop low-lactose or lactose-free products. Thus, sugar enzymatic hydrolysis from the *β*-galactosidase enzyme, generating glucose and galactose monosaccharides, emerges as a key biotechnological process with application in the dairy industry. *β*-Galactosidase was first used in the preliminary treatment of milk used in the production of candies and fermented dairy products. This application leads to an enhancement of the sensorial and technological properties of the end products, such as higher sweetness, higher solubility, and decrease in fermentation period [11, 12].

Another application for the hydrolysis process aims at adding value to whey, whose improper disposal poses a serious environmental problem. The number of microorganisms capable of metabolizing lactose as a source of carbon is smaller than the number of microorganisms capable of metabolizing glucose and galactose [13]. Therefore, whey lactose enzymatic hydrolysis makes the bioremediation processes and biomolecules and biomass productions viable with the use of these kinds of whey as cultivation media [14, 15].

*β*-Galactosidases have animal, vegetable, or microbial (bacterium, fungus, and yeast) origins, but the microbial enzymes show a higher productivity, resulting in cost reduction. The *β*-galactosidases used in industrial scale must come from* Generally Recognized as Safe* (GRAS) microorganisms. Enzymes obtained from fungi (*Aspergillus oryzae* and* Aspergillus niger*) and yeasts (*Kluyveromyces lactis* and* Kluyveromyces fragilis*) show great commercial potential. The choice of the *β*-galactosidase source usually depends on the hydrolysis reaction conditions. The ones from fungi present an optimum pH between 2.5 and 5.4, being usually used for acid whey hydrolysis. The ones obtained from yeasts present optimum pH between 6.0 and 7.0, and its use is more adequate for milk and sweet whey lactose hydrolysis. Other important features for the use of the enzyme in industrial processes include thermostability and the elevated enzymatic activity at low temperature, which enables its application in conditions that do not alter the sensorial and nutritional features of milk and its derivatives [11, 16].

In this context, the work aimed at evaluating the influence of enzyme concentration, temperature, and reaction time in the lactose hydrolysis process in milk, cheese whey, and whey permeate, using two commercial *β*-galactosidases of microbial origins.

## 2. Material and Methods

### 2.1. Material

The low fat powdered milk and the powdered cheese whey used for this paper were ceded by Brasil Foods S.A. (Teutônia, Rio Grande do Sul, Brazil). The cheese whey powder was provided by Arla Foods (Porteña, Córdoba, Argentina). The commercial *β*-galactosidases enzymes used, Lactomax F30 and Lactomax Pure, obtained from the* Aspergillus oryzae* (*A. oryzae*) and* Kluyveromyces lactis* (*K. lactis*) strains, respectively, were provided by Prozyn (Butantã, São Paulo, Brazil).

### 2.2. *β*-Galactosidase Assay

The *β*-galactosidase activities of the commercial enzymes were assayed using o-nitrophenyl-*β*-D-galactopyranoside (ONPG) as substrate. The initial rate of formation of free o-nitrophenol (ONP) was recorded spectrophotometrically at 415 nm. One unit of enzyme activity (U) is defined as the amount of enzyme that liberates 1.0 *μ*mole of o-nitrophenol per minute under assay conditions.

### 2.3. Milk and Dairy Whey Enzymatic Hydrolysis Process

We added 100 mL of low fat milk, cheese whey, or whey permeate reconstructed at 5% (m/v) of lactose to 250 mL Erlenmeyer flasks. The processes of enzymatic lactose hydrolysis were performed using 3, 6, and 9 U/mL concentrations for the* A. oryzae* or* K*.* lactis* *β*-galactosidase in orbital rotation shaker (MA 830, Marconi, Piracicaba, SP, Brazil), at 150 rpm, with temperatures of 10 and 55°C for the* A. oryzae* enzyme and 10 and 37°C for the* K*.* lactis* *β*-galactosidase. These enzymatic concentrations corresponded to 0.1, 0.2, and 0.3 g/L of* A. oryzae* *β*-galactosidase and to 0.37, 0.74, and 1.11 g/L of* K*.* lactis* *β*-galactosidase. After 0, 1, 2, 4, 8, and 12 h of hydrolysis reaction, we collected samples that were submitted to a 100°C for 10 min, so as to inactivate the enzyme, and afterwards we determined the glucose concentration. All the experiments were performed in triplicate.

We calculated the milk and dairy whey lactose hydrolysis efficiency (*E*_*h*_) according to the following:

(1) $$\begin{array}{c} E_{h}\left(\;\%\;\right)=\frac{C_{glu}\times {MM}_{lac}}{{Ci}_{lac}\times {MM}_{glu}}\times 100, \end{array}$$

where *C*_glu  _ is glucose concentration, MM_lac_ is molar mass of lactose, *Ci*_lac_ is initial lactose concentration (50 g/L), and MM_glu_ is molar mass of glucose.

### 2.4. Determination of Glucose Concentration

The glucose concentration was measured with the oxidase-peroxidase colorimetric method, using the enzymatic kit for glucose determination (Labtest, MG, Brasil) and absorbance reading in spectrophotometer (Genesys 10S UV-Vis, Thermo Scientific, USA) at 505 nm. The glucose concentration was established based on a calibration curve. The analyses were performed in triplicate.

### 2.5. Statistical Analysis

The glucose concentration results represent the average from three independent experiments, performed in triplicate. The statistical evaluation of the conversion rate of lactose in glucose, present in milk, cheese whey, and permeate, in the different conditions of enzymatic hydrolysis, was performed through the Analysis of Variance (ANOVA), and the significance of the model was verified by the *F* test. In the significant models, the averages were compared to the Tukey test, in a significance level of 95% (*p* < 0.05) using the BioEstat 5.0 software.

## 3. Results and Discussion

**Figure 1:**
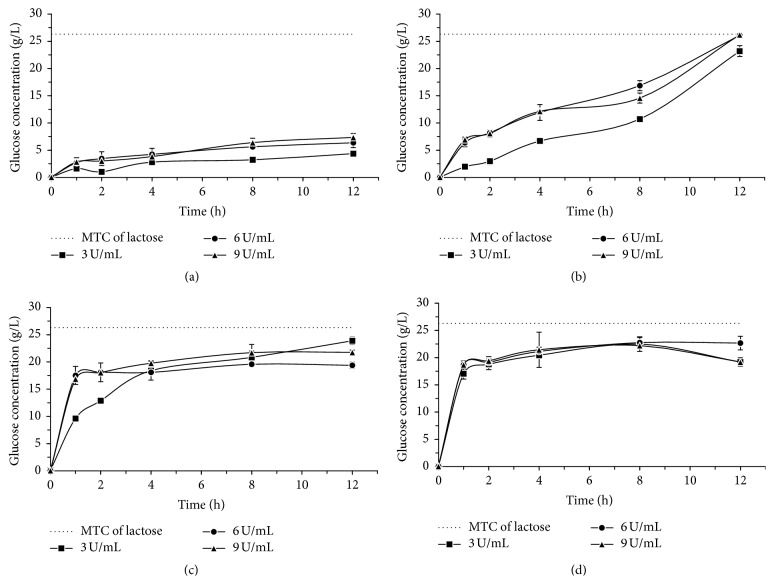
Glucose concentration from the enzymatic lactose hydrolysis of milk using* A. oryzae* *β*-galactosidase, (a) 10°C, (b) 55°C, and* K. lactis* *β*-galactosidase, (c) 10°C, (d) 37°C. The dotted line in each graph indicates the corresponding glucose concentration to maximum theoretical conversion (MTC) of lactose.

**Figure 2:**
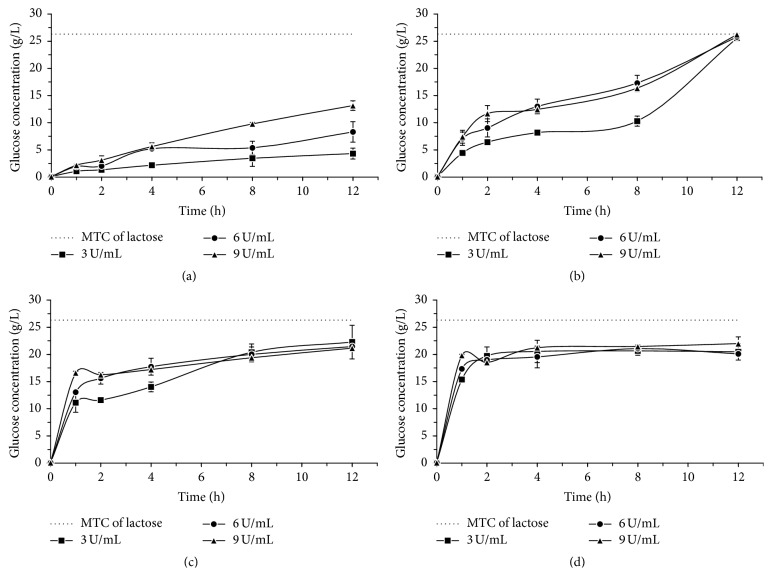
Glucose concentration from the enzymatic lactose hydrolysis of cheese whey using* A*.* oryzae* *β*-galactosidase, (a) 10°C, (b) 55°C, and* K. lactis* *β*-galactosidase, (c) 10°C, (d) 37°C. The dotted line in each graph indicates the corresponding glucose concentration to maximum theoretical conversion (MTC) of lactose.

**Figure 3:**
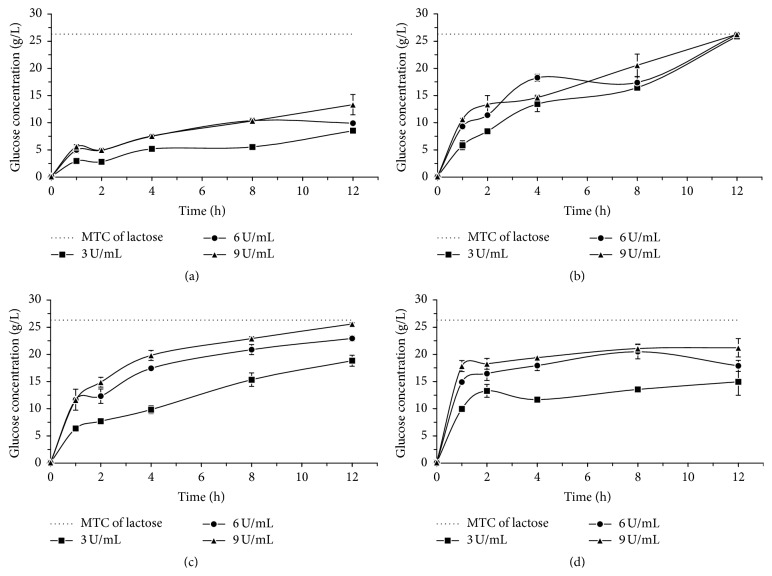
Glucose concentration from the enzymatic lactose hydrolysis of whey permeate using* A*.* oryzae* *β*-galactosidase, (a) 10°C, (b) 55°C, and* K. lactis* *β*-galactosidase, (c) 10°C, (d) 37°C. The dotted line in each graph indicates the corresponding glucose concentration to maximum theoretical conversion (MTC) of lactose.

Figures 1, 2, and 3 show results from the lactose hydrolysis of milk, cheese whey, and permeate, respectively, using the* A. oryzae* (at 10 and 55°C) and* K. lactis* enzymes (at 10 and 37°C), with a 12 h reaction. The enzymatic concentrations used were 3, 6, and 9 U/mL (one unit of *β*-galactosidases activity (U) was defined as the amount of enzyme that liberates 1.0 *μ*mole of o-nitrophenol per minute under assay conditions).

The lactose hydrolysis processes of milk, whey, and permeate were performed at low temperature (10°C) for both enzymes, with the aim of not altering the sensorial and nutritional characteristics of the milk and dairy whey. The enzymatic optimum temperature effect of each commercial *β*-galactosidase,* A. oryzae* (55°C), and* K. lactis* (37°C) was also evaluated in the hydrolysis process.

According to Campos et al. [17], the *β*-galactosidase enzyme has been used at temperatures under optimum value of reaction, in order to avoid bacterial multiplication in the milk, as temperatures around 37°C correspond to the optimum condition for the development of various deteriorative and pathogenic microorganisms. The enzymes activity is influenced by multiple environmental factors, among which temperature is one of the most important parameters, and may affect the tridimensional structure or the protein conformation [18].

Comparing the hydrolysis process at 10°C and at the optimum temperature of each enzyme, we verified that, under the experimental conditions applied, the temperature variation effect was more expressive for* A. oryzae* *β*-galactosidase than for* K. lactis* *β*-galactosidase. Figures 1, 2, and 3 show that the lactose hydrolysis of milk, whey, and permeate, respectively, with the fungal enzyme was higher at the optimum temperature in relation to the one at 10°C; yet for the* K. lactis* enzyme, the glucose concentrations generated from the lactose hydrolysis are similar under both temperatures (10 and 37°C), regardless of the raw material.

Regarding the enzyme concentration used in the lactose hydrolysis process, we verified that the variation for this parameter had higher influence when the* A. oryzae* *β*-galactosidase was used, regardless of the studied raw material. For the* K. lactis* enzyme, the lactose hydrolysis results only presented the same behavior of the fungal *β*-galactosidase when the whey permeate was used as a substrate. The results were similar for the milk and cheese whey hydrolysis, regardless of the tested* K. lactis* enzyme concentration.

Enzyme concentration directly influences lactose hydrolysis, as was already observed in other papers [19, 20]. Horner et al. [19] evaluated the lactose hydrolysis of raw and pasteurized milk using four commercial* Kluyveromyces* *β*-galactosidases at 2°C for 72 h. The authors observed that the fourfold increase in the enzyme concentration doubled the hydrolyzed lactose concentration in the milk after a 12 h reaction.

Akgül et al. [20] analyzed the lactose hydrolysis of low fat milk using a commercial* K. lactis* *β*-galactosidase in concentrations around 3, 6, 9, and 12 U/mL at 37°C for 30 h. The authors verified that the increase of the enzymatic concentration led to a conversion increase in the lactose present in the milk.

In Figures 1, 2, and 3, we observed that for* A. oryzae* *β*-galactosidase the concentrations of glucose gradually increased throughout the whole hydrolysis period, reaching, in 12 h at optimum temperature, values close to the maximum theoretical conversion of lactose in its respective sugars. As for the* K. lactis* *β*-galactosidase, we verified a gradual increase in glucose concentration during the first two hours of reaction, and after that, it remained practically constant throughout the evaluated process, reaching values of about 20 g/L of glucose.

Tables 1, 2, and 3 show the efficiency results for the lactose hydrolysis of milk, cheese whey, and permeate, respectively, using the* A. oryzae* (at 10 and 55°C) and* K. lactis* enzymes (at 10 and 37°C) after 2 h of reaction. The enzymatic concentrations used were 3, 6, and 9 U/mL.

The hydrolysis efficiency was evaluated in 2 h reaction taking into account the possible industrial application of the enzymes in the referred process.

No significant difference (*p* < 0.05) was observed between the efficiencies of the lactose hydrolysis in milk, whey, and permeate when using 6 and 9 U/mL concentrations, for both enzymes at optimum temperatures and at 10°C. However, for the 3 U/mL concentration, efficiency was significantly lower (*p* < 0.05) for most of the conditions evaluated.

During 2 h of reaction, for the same enzyme concentration, the* K. lactis* *β*-galactosidase presented higher hydrolysis efficiency than the* A. oryzae* *β*-galactosidase. This higher lactose conversion rate was verified even when the* K. lactis* enzyme was used at 10°C and the* A. oryzae* one at the optimum reaction temperature.

We observed a higher milk and whey lactose hydrolysis efficiency when using the* K. lactis* *β*-galactosidase and in the permeate when using the* A. oryzae* *β*-galactosidase. These results probably occurred due to the different pH values of the raw materials. The pH values of reconstructed milk and whey used in this study presented values of about 6.8 and 6.4, respectively. The whey permeate presented a pH value of around 5.9. The* A. oryzae* *β*-galactosidase used presents an optimum pH in a 4.5–5.0 range, and the one from* K. lactis* presents pH between 7.3 and 7.7.

When using the* A. oryzae* *β*-galactosidase in 6 and 9 U/mL concentrations, we verified a total lactose hydrolysis in milk, whey, and permeate at 55°C at the end of 12 h of enzymatic reaction (Figures 1(b), 2(b), and 3(b)). The hydrolysis rate obtained in the present study was higher than the one described by Haider and Husain [21], who evaluated the lactose hydrolysis process in milk and whey using the* A. oryzae* *β*-galactosidase, at 37°C, in 0.88 and 0.44 U/mL concentrations, respectively. The authors observed a maximum hydrolysis of 61% for milk after 4 h of reaction and of 70% for whey after 3 h of reaction.

The maximum lactose hydrolysis efficiencies, in 2 h of reaction, were obtained using a* K. lactis* *β*-galactosidase at 37°C (Tables 1, 2, and 3). In the milk, the highest conversion rate observed was 73.84% with 9 U/mL of enzymes. For the whey, the maximum hydrolysis efficiency was 74.98% with 3 U/mL of enzymes. As for the permeate, it was 69.42% with 9 U/mL of enzymes. For the* K. lactis* *β*-galactosidase, in none of the conditions evaluated, a 100% hydrolysis efficiency was reached (Figures 1(c), 1(d), 2(c), 2(d), 3(c), and 3(d)). However, the obtained results may be considered as satisfactory, since, according to Hourigan [22], the lactose concentrations reduction from 70 to 80% in dairy products is enough for most people who are lactose intolerant.

Higher milk lactose hydrolysis values using the* Kluyveromyces* enzyme are described in the literature. Horner et al. [19], using commercial* Kluyveromyces* *β*-galactosidases in 3, 6, 9, and 12 U/mL concentrations, at 2°C after 48 h of reaction, verified values of approximately 100% lactose hydrolysis efficiency for pasteurized milk. Antunes et al. [23] evaluated the physicochemical, microbiological, and sensorial characteristics of delactosed milk using 20 U/mL of the* K. lactis* *β*-galactosidase enzyme at 10°C. Under these conditions, the authors verified a hydrolysis rate of 90% in 21 h of reaction.

## 4. Conclusion

The results obtained indicate that at temperature of 10°C the* K. lactis* *β*-galactosidase enzyme is more efficient in the milk, cheese whey, and whey permeate lactose hydrolysis when compared to* A. oryzae*. However, in the enzyme reaction time and concentration conditions evaluated, 100% lactose hydrolysis was not reached using the* K. lactis* *β*-galactosidase. The total lactose hydrolysis in whey and permeate was obtained with the* A. oryzae* enzyme, when using its optimum temperature (55°C), at the end of 12 h of reaction, regardless of the enzyme concentration used. For milk lactose, this result occurred in the concentrations of 6 and 9 U/mL, with the same time and temperature conditions.

## Figures and Tables

**Table 1:** Efficiency of milk lactose hydrolysis using *A. oryzae* and *K. lactis β*-galactosidases in 3, 6, and 9 U/mL concentrations and 2 h of reaction.

Enzyme conc. (U/mL)	*Aspergillus oryzae*				*Kluyveromyces lactis*			
	10°C		55°C		10°C		37°C	
	*C*_glu_ (g/L)	*E*_*h*_ (%)	*C*_glu_ (g/L)	*E*_*h*_ (%)	*C*_glu_ (g/L)	*E*_*h*_ (%)	*C*_glu_ (g/L)	*E*_*h*_ (%)
3	1.04^b^	3.96^b^	2.98^b^	11.32^b^	12.88^b^	48.94^b^	18.87^a^	71.70^a^
6	3.45^a^	13.12^a^	8.21^a^	31.20^a^	18.08^a^	68.70^a^	19.15^a^	72.78^a^
9	3.02^a^	11.48^a^	8.08^a^	30.70^a^	18.05^a^	68.60^a^	19.43^a^	73.84^a^

^ab^Means with different superscripts in the same column are significantly different, according to Tukey test (*p* < 0.05).

**Table 2:** Efficiency of cheese whey lactose hydrolysis using *A. oryzae* and *K. lactis β*-galactosidases in 3, 6, and 9 U/mL concentrations and 2 h of reaction.

Enzyme conc. (U/mL)	*Aspergillus oryzae*				*Kluyveromyces lactis*			
	10°C		55°C		10°C		37°C	
	*C*_glu_ (g/L)	*E*_*h*_ (%)	*C*_glu_ (g/L)	*E*_*h*_ (%)	*C*_glu_ (g/L)	*E*_*h*_ (%)	*C*_glu_ (g/L)	*E*_*h*_ (%)
3	1.34^b^	5.10^b^	6.43^b^	24.44^b^	11.58^b^	44.00^b^	19.73^a^	74.98^a^
6	2.04^ab^	7.76^ab^	9.04^ab^	34.36^ab^	15.61^a^	59.32^a^	18.99^a^	72.16^a^
9	3.12^a^	11.86^a^	11.68^a^	44.38^a^	16.25^a^	61.76^a^	18.51^a^	70.34^a^

^ab^Means with different superscripts in the same column are significantly different, according to Tukey test (*p* < 0.05).

**Table 3:** Efficiency of whey permeate lactose hydrolysis using *A. oryzae* and *K. lactis β*-galactosidases in 3, 6, and 9 U/mL concentrations and 2 h of reaction.

Enzyme conc. (U/mL)	*Aspergillus oryzae*				*Kluyveromyces lactis*			
	10°C		55°C		10°C		37°C	
	*C*_glu_ (g/L)	*E*_*h*_ (%)	*C*_glu_ (g/L)	*E*_*h*_ (%)	*C*_glu_ (g/L)	*E*_*h*_ (%)	*C*_glu_ (g/L)	*E*_*h*_ (%)
3	2.82^b^	10.72^b^	8.42^b^	32.00^b^	7.70^b^	29.26^b^	13.28^b^	50.46^b^
6	4.93^a^	18.74^a^	11.38^a^	43.24^a^	12.31^a^	46.78^a^	16.46^a^	62.54^a^
9	4.96^a^	18.84^a^	13.35^a^	50.74^a^	14.38^a^	54.64^a^	18.27^a^	69.42^a^

^ab^Means with different superscripts in the same column are significantly different, according to Tukey test (*p* < 0.05).
